# Pore structure characterization of Chang-7 tight sandstone using MICP combined with N_2_GA techniques and its geological control factors

**DOI:** 10.1038/srep36919

**Published:** 2016-11-10

**Authors:** Zhe Cao, Guangdi Liu, Hongbin Zhan, Chaozheng Li, Yuan You, Chengyu Yang, Hang Jiang

**Affiliations:** 1 State Key Laboratory of Petroleum Resources and Prospecting, China University of Petroleum, Beijing 102249 PR, China; 2 College of Geosciences, China University of Petroleum, Beijing, 102249 PR, China; 3 Department of Geology and Geophysics, Texas A&M University, College Station, TX 77843-3115, USA; 4 Research Institute of Petroleum Exploration and Development of Changqing Oilfield Company, PetroChina, Xi’an 710018 PR, China; 5 Strategic Research Center of Oil and Gas Resource, Ministry of Land and Resources, Beijing 100034 PR, China

## Abstract

Understanding the pore networks of unconventional tight reservoirs such as tight sandstones and shales is crucial for extracting oil/gas from such reservoirs. Mercury injection capillary pressure (MICP) and N_2_ gas adsorption (N_2_GA) are performed to evaluate pore structure of Chang-7 tight sandstone. Thin section observation, scanning electron microscope, grain size analysis, mineral composition analysis, and porosity measurement are applied to investigate geological control factors of pore structure. Grain size is positively correlated with detrital mineral content and grain size standard deviation while negatively related to clay content. Detrital mineral content and grain size are positively correlated with porosity, pore throat radius and withdrawal efficiency and negatively related to capillary pressure and pore-to-throat size ratio; while interstitial material is negatively correlated with above mentioned factors. Well sorted sediments with high debris usually possess strong compaction resistance to preserve original pores. Although many inter-crystalline pores are produced in clay minerals, this type of pores is not the most important contributor to porosity. Besides this, pore shape determined by N_2_GA hysteresis loop is consistent with SEM observation on clay inter-crystalline pores while BJH pore volume is positively related with clay content, suggesting N_2_GA is suitable for describing clay inter-crystalline pores in tight sandstones.

Influenced by increasing demand of global energy, unconventional reservoirs had received increasing attention in the past decade due to its enormous exploration potential. With the successful application of horizontal drilling and multistage hydraulic fracturing technology, tight oil such as Bakken oil (Williston basin, North America) and Eagle Ford oil (South Texas, USA) had been exploited commercially. It had become the focus of research in global petroleum geology^1^ and was considered as the most practical unconventional replacement for oil and gas besides shale gas^2^. Tight sandstones and shale had stored a considerable amount of hydrocarbon in their structures. Only by understanding the structure of pore networks can engineers and geologists make more accurate predictions of producible gas/oil and optimize its extraction^3^,^4^,^5^. Contrast to conventional hydrocarbon resources, the pore structure of tight reservoirs was difficult to characterize because of the ultra-fine pore throat, generally broad pore size distribution (PSD) with significant portion being nanoscale^6^,^7^. Washburn (1921) presented a capillary tube model, laid theoretical foundation for porous material pore structure determination using mercury intrusion capillary pressure (MICP) method^8^. This model assumed that porous media were composed of capillary tubes having different diameters and pore size distribution which can be calculated according to a pressure–volume curve produced by mercury intrusion process. Being able to measure a wide range of pore sizes, having short measuring times and acquiring abundant information about pore structure^9^, MICP had been a viable tool for the characterization of reservoir rocks for over six decades^10^,^11^. It was one of the most used methods to measure the porosity and PSD in porous materials^12^ since Ritter and Drake (1945) proposed this technique at first^13^.

Wardlaw and Cassan (1979) measured the grain particle size, pore size, and pore-throat size for 27 sandstone samples from various locations worldwide and of various geologic ages^14^. Core depths ranged from 1000 to 3000 m (3280 to 9840 ft). This sample set was chosen to represent sandstones with permeability values greater than 1 × 10^−3^ μm^2^. The pore-throat size was determined by MICP at threshold entry pressure and at 50% mercury saturation. The result showed that pore size and throat radius of these sandstones exhibited a downward trend with particle reducing from median sand to coarse silt while most samples had a median throat radius greater than 1 μm^14^.

However, tight reservoirs usually had low air permeability (lower than 0.1 × 10^−3^ μm^2^)^15^. Most tight sandstones (such as Lower Cretaceous Travis Peak Formation in East Texas Basin and Upper Cretaceous Mesaverde Formation in Piceance Basin) had median throat radius lower than 100 nm^6^. As research on reservoir rocks extended to nano scale pore sizes gradually, MICP exhibited some limitations on measurement range. Mercury intrusion was good at calculating PSD of pores between 30 nm and 200 mm and high-pressure mercury intrusion can detect pores from 3 nm to 200 mm^9^,^16^. Some studies suggested that mercury intrusion data collected at pressures above about 70 MPa were probably not meaningful because of the pore structure changes, such as possible particle breakdown, the opening of otherwise closed pores^9^,^17^ and some pore structure distortion occurring at the lower pore size limit (~3 nm)^7^,^18^.

Washburn equation assumed the pore configuration to be an unconnected network of cylindrical pores. As a result, PSD was regarded as an equivalent capillary. Another common limitation of MICP was related to the ink-bottle configuration, i.e., pore geometries composed of a system of large chambers (body pores) interconnected by narrow pores (throats). In such a system pore network effects were assumed to be the most important reason for the large hysteresis between intrusion and extrusion in MICP experiments^19^. Meanwhile over-estimation of the smaller pores (throat) in detriment of the larger ones^11^ might shift the pore size distribution in the direction of the smaller pores.

N_2_ physisorption, which primarily acquired data within relative pressure (the ratio of gas equilibrium pressure to saturated vapor pressure) range of 0.01–1, was one of the most widely used experimental methods for quantitative pore structure characterization of porous materials, such as calculating PSD, specific surface area (SSA) and pore volume (PV)^9^,^18^,^20^. According to the IUPAC recommendations^21^, pores were classified according to their diameter sizes as micropores (less than 2 nm), mesopores (2–50 nm) and macropores (greater than 50 nm). The classical pore size model developed by Barret, Joyner and Halenda (BJH)^22^, which was based on the Kelvin equation and corrected for multilayer adsorption, was most widely used for calculations of PSD over the mesopores (2–50 nm) and a portion of macropores (50–300 nm)^23^,^24^.

Low-pressure nitrogen (N_2_) gas adsorption (N_2_GA) was only applicable to pores whose diameters were less than 300 nm^9^,^18^,^20^,^25^. Both MICP and N_2_GA had some limitations in characterizing pore structure of tight oil reservoirs. To characterize the overall PSD of tight oil reservoirs, a combination of these two invasion methods appeared to be necessary^26^. One of the advantages of combining MICP and N_2_GA was to obtain the overall pore size distribution, which was particularly useful for the tight sandstones of this study. This combinational analysis had been widely applied to quantitative evaluation of nano matrix pore in coals^27^,^28^,^29^ and had been gradually proven to be an effective method to characterize pore structure in shales with the rise of unconventional reservoir research^18^,^25^,^30^.

However, there were only a few reports on the pore structure characteristics of tight gas sandstone^18^ and nearly none study on tight oil sandstone using the combined MICP and N_2_GA approach. One of the objectives of this work was to understand the pore structure and its geological significance of tight sandstones using an approach combining the advantages of MICP and N_2_GA, based on Chang-7 sandstone samples in Longdong Area, Ordos Basin of China. Discussion about relationship between pore radii (micron-size or nano-size) and pore types (residual primary intergranular pores, dissolved pores or inter-crystalline pores) in tight sandstones were presented as well. In addition, microscopic observation, mineral composition and grain size analysis were applied to establish relationships between geological factors and pore structure parameters in tight sandstones.

Upper Triassic Yanchang Formation was a set of terrigenous detrital rocks formed in a sustained subsidence process, dominated by alluvial fan, alluvial plain, deltaic, fluvial and lacustrine complexes^27^,^31^. For exploration and exploitation purposes, it was subdivided into 10 informal subsections named Chang-10 to Chang-1 from the bottom to the top by marker beds, sedimentary cycles or lithological association (Fig. 1B)^32^.

The Chang-7 subsection was deposited at the maximum lake transgression period when a set of dark mudstone and oil shale of deep lacustrine facies were widely distributed as dominant high quality hydrocarbon source rock for most of Mesozoic oil–gas reservoirs in Ordos Basin^33^. Extensive sandy debris flow and turbidite deposit sand body were interbedded with source rock, constituting the key target of tight oil exploration and exploitation area in Ordos Basin^34^.

The study area lied in Longdong District, covering Qingyang, Zhenjing and Huachi area in Ordos Basin (Fig. 1A). The Chang-7 sands in this area were mainly gravity flow sediments in delta front and semi-deep to deep lacustrine facies. It was mainly made up of lithic arkose and feldspathic litharenite characterized by fine grain size and high matrix content^34^.

## Results

### Lithology, mineral composition, and grain size and porosity

**Figure 1:**
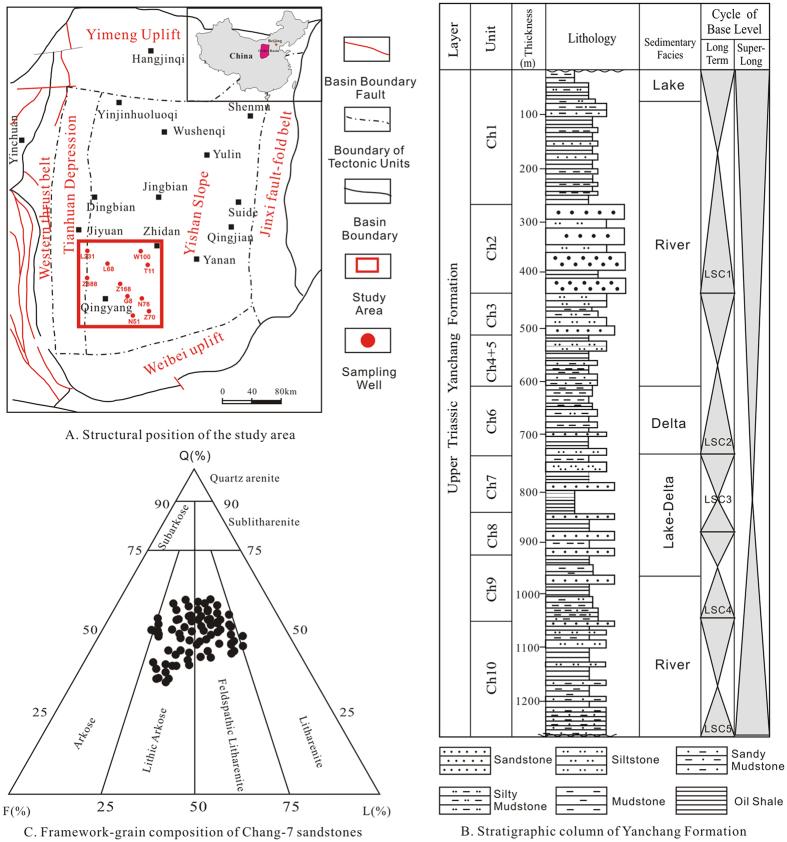
(**A**) Location of study area in Ordos Basin and sampling well locations are shown (modified from Lai *et al*.)^52^. (**B**) Stratigraphic column of Upper Triassic Yanchang (Ch) Formation in Ordos Basin (modified from Lai *et al*.)^52^. (**C**) Triangular ternary diagram showing the framework-grain composition of Chang-7 sandstones in study area.

**Figure 2:**
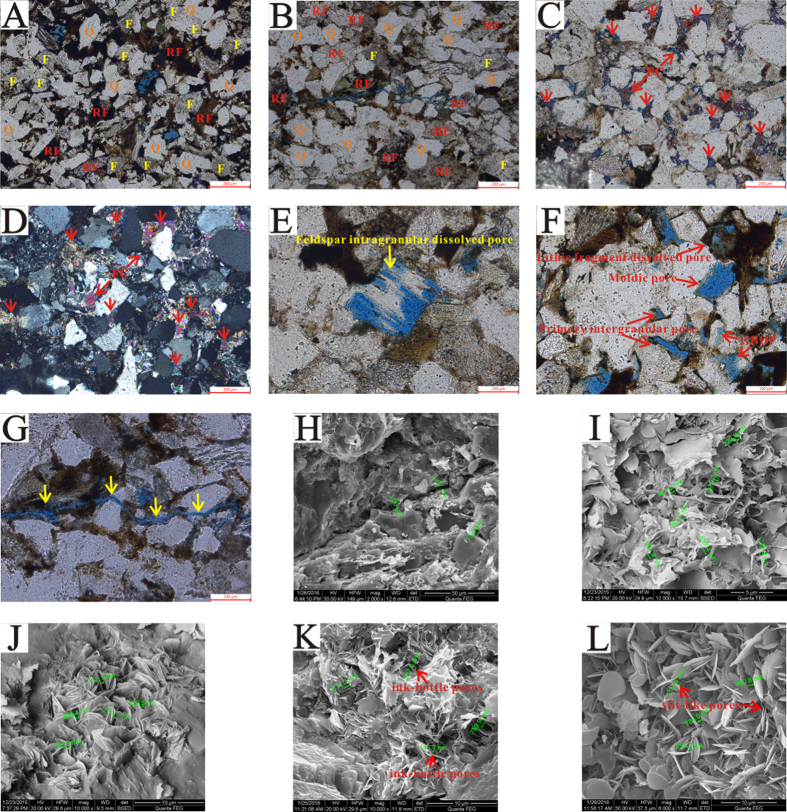
Lithology characteristics and pore systems of Chang-7 tight reservoirs in Ordos Basin. (**A**) Wu100, 1995.86 m, fine-grained, subrounded to subangular, poorly sorted lithic arkose. Q-quartz; F-feldspar; RF-rock fragment. (**B**) Zhen388, 2021.6 m, fine-grained, subrounded to subangular, moderately sorted feldspathic litharenite. (**C,D**) Ning76, 1724.88 m, Fe-calcites (FC) cement (red arrow signified) is the most common pore-filling constituents, plane-polarized light and perpendicular-polarized light, thin section colored by sodium alizarinsulfonate. (**E**) Ning76, 1776.95 m, intragranular pore caused by feldspar dissolution, blue casting thin section. (**F**) Wu100, 2008.29 m, residual primary intergranular pore, lithic fragment dissolved pore, moldic pore and feldspar grain boundary dissolved pore, blue casting thin section. (**G**) Zhen388, 2021.6 m, thin section, micro fractures. (**H**) Zhen168, 1777.77 m, SEM image, micro fractures. (**I**) Wu100, 1968 m, ink-bottle shape inter-crystalline pores developed in silk and roll form illite, pore diameter mainly ranges 100 to 400 nm. (**J**) Wu100, 1995.86 m, slit-like inter-crystalline pores developed in lamellated and plate-like chlorite, pore diameter mainly ranges 100 to 400 nm. (**K**) Z388, 1907.4 m, ink-bottle shape intercrystalline pores developed in illite-smectite mixed-layer. (L) L231, 2069.76 m, narrow slit-like pores in interspace of chlorite aggregates.

**Figure 3:**
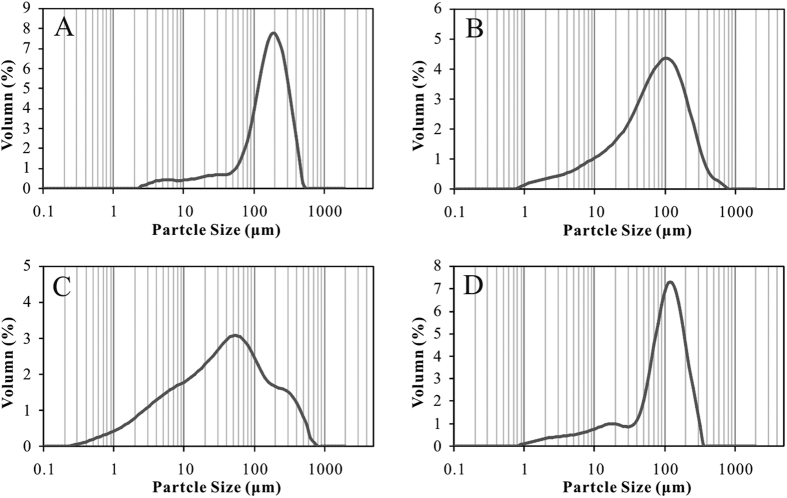
Representative grain size distribution profiles of Chang-7 tight sandstones. (**A**) N76, 1724.88 m. (**B**) N76, 1742.8 m. (**C**) T11, 1441.35 m. (**D**) Z388, 1907.4 m.

The lithologies of Chang-7 sandstones in study area were mainly composed of lithic arkose and feldspathic litharenite according to Folk’s classification scheme^35^ (Fig. 1C and Fig. 2A,B) based on analyses of more than 200 thin sections. Interstitial material included matrix with an average content of 13.51% and cement with the value of 7.88% (identified by microscopic observation) dominated by carbonate, silicate, illite and chlorite minerals (Fig. 2C,D). Qualitative and quantitative analyses by X-ray diffraction (XRD) were performed and the results showed that components of the Chang-7 sandstone in study area were composed of clay minerals, quartz, feldspar, calcite, and dolomite. Quartz + feldspar were the major components that ranged from 50% to 80%, while clay minerals ranged from 10% to 40% (Supplementary Table S1). Carbonate minerals contents varied greatly from trace to 40% with an average content less than 20%. The porosity was low overall, ranging from 1.20% to 13.98%, with an average value of 8.09%. The median grain size (d(0.5)) ranged from 34.15 to 155.52 μm with an average of 102.52 μm. The grain size distribution of most samples revealed a predominantly fine size fraction (Supplementary Table S2)^36^, but the overall distribution spanned from clay to sand grain size fractions (Fig. 3). The standard deviation (*σ*_1_) ranged from 1.26 to 2.38 with an average of 1.61, indicating that Chang-7 sandstone in study area was poorly sorted^37^.

### Pore types and characteristics

Thin section and SEM observation revealed the presence of four types of pores in the Chang-7 tight sandstone reservoirs, including residual primary intergranular pores, dissolved pores, inter-crystalline pores and micro-fractures.

Residue intergranular pores, mostly triangular or polygonal in shape (Fig. 2F), were affected by compaction or cementation generally. This kind of pores ranged between 10 μm and 70 μm and had straight pore edge and clear grain boundary without any dissolution. In spite of the fact that Chang-7 sandstones were characterized by fine grain size, high matrix content and strong compaction, residue intergranular pores were still one of the main pore types in the study area.

Dissolved pores were the primary pore spaces contributor for Chang-7 sandstones (identified by thin section), mainly associated with feldspar dissolution, which can enlarge the intergranular pores or form new intragranular pores. Dissolved pores relevant with carbonate cement were rare, for the posterior formation time (later than organic acid dissolution) of carbonate cement in this area^34^. Feldspar dissolved pores included intragranular dissolved pores and grain boundary dissolved pores, according to different dissolution position (Fig. 2E,F). In addition, some moldic pores and lithic fragment dissolved pores were observed as well. This kind of pores ranged between 30 μm and 100 μm and presented various irregular shapes, characterized by relatively large pore sizes and good connectivity.

Inter-crystalline pores were often associated with clay minerals, mainly including illite and chlorite, ranging from 50 nm to 2 μm, with the majority between 100 nm and 400 nm (Fig. 2I,J). These pores were one of the most important pore types and ubiquitous under SEM observation in Chang-7 tight sandstones.

Micro-fractures were also identified in some samples, the widths of the micro fractures ranged from 2 μm to 10 μm, with the majority between 2 μm and 5 μm, while their lengths were up to 10 mm (Fig. 2G,H).

### Pore throat structure determination using MICP

**Figure 4:**
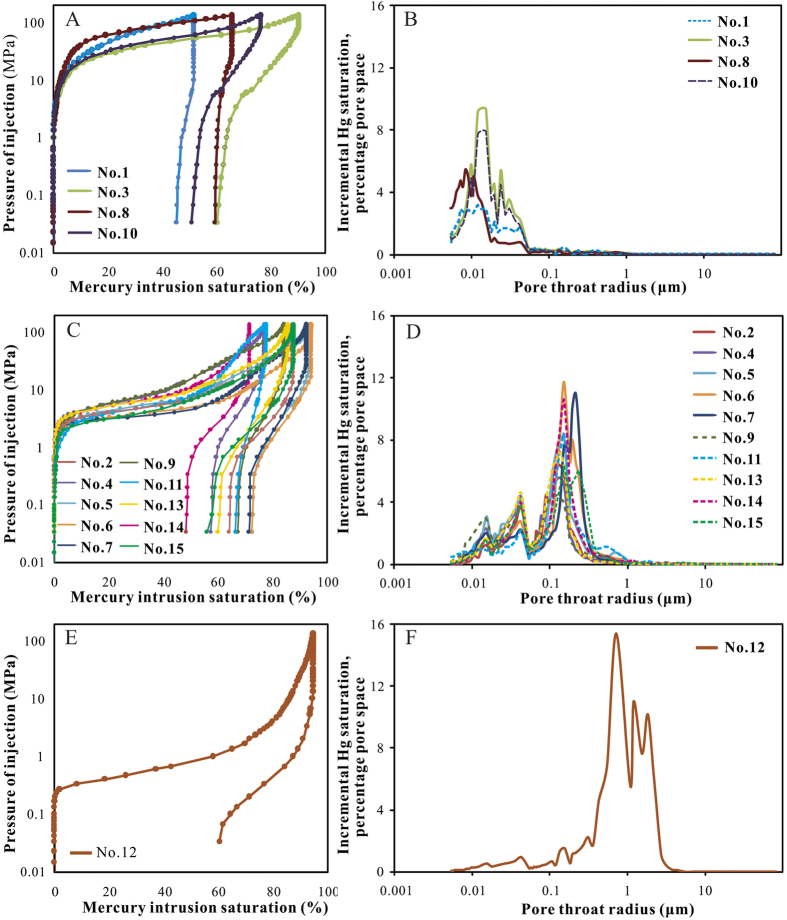
MICP curves (**A,C,E**) and pore size distribution by MICP (**B,D,F**) of Chang-7 samples. (**A,B**)- group I. (**C,D**)- group II. (**E,F**)- group III.

As indicated by MICP analyses, Chang-7 tight sandstone reservoirs in study area were generally characterized by small pore throats, high capillary pressure and a median pore throat radius of less than 1 μm. The MICP curves of the samples (Fig. 4 and Supplementary Table S3) showed that the maximum pore throat radius (*r*_*max*_) was 0.027–2.163 μm and the average value was 0.329 μm. The median pore throat radius (*r*_50_) was between 0.006 μm and 0.910 μm with an average value of 0.127 μm. Overall, the pore throats were quite small. The sorting coefficients of the pore throats were around 1.15–2.29 with an average sorting of 1.55 (sorting coefficient was inversely proportional to the sorting quality). The displacement pressure (*P*_*d*_) was between 0.34 MPa and 27.2 MPa with an average value of 6.48 MPa while the median capillary pressure (*P*_*c*50_) ranged from 0.84 MPa to 125.21 MPa with an average value of 28.93 MPa, suggesting that oil or gas cannot enter into the reservoirs easily.

**Figure 5:**
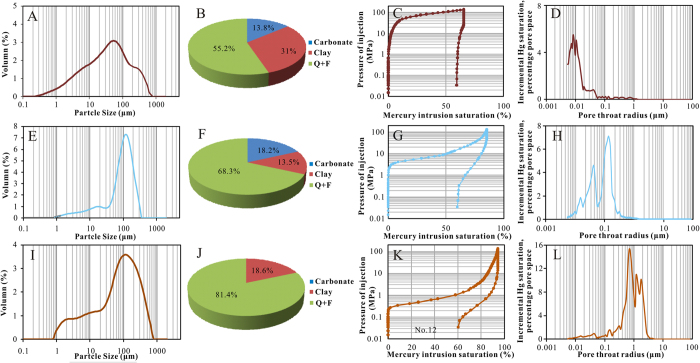
Grain size distribution, mineral composition, MICP curves and pore size distribution of selected representative samples of three groups. It indicated that the sample with less carbonate/clay content and coarser grain size is tend to have larger pore throat radius and less displacement pressure. (**A–D**) - No. 8, T11, 1441.35 m; (**E–H**) - No. 13, Z388, 1907.4 m; (**I–L**) - No. 12, W100, 2008.29 m.

These samples can be divided into three groups according to differences presented on pore size distribution and some other pore structure parameters such as displacement pressure, capillary pressure mid-value and pore throat radius mid-value. These three groups included: (I) extremely fine pore throat (mean *r*_*max*_ of 0.049 μm) and very high capillary pressure (mean *P*_*d*_ of 16.83 MPa, mean *P*_*c*50_ of 85.64 MPa) (Fig. 4A,B); (II) fine pore throat (mean *r*_*max*_ of 0.257 μm) and high capillary pressure (mean *P*_*d*_ of 2.96 MPa, mean *P*_*c*50_ of 9.05 MPa) (Fig. 4C,D); (III) moderate pore throat (mean *r*_*max*_ of 2.163 μm) and moderate capillary pressure (mean *P*_*d*_ of 0.34 MPa, mean *P*_*c*50_ of 0.84 MPa) (Fig. 4E,F). It was noteworthy that the extremely fine pore throat samples always had relatively high carbonate minerals content or fine grain size while relatively better pore throat sample had less carbonate minerals and coarser grain size (Fig. 5). Related contents were illustrated in subsequent sections.

### Pore structure determination using N_2_GA

**Figure 6:**
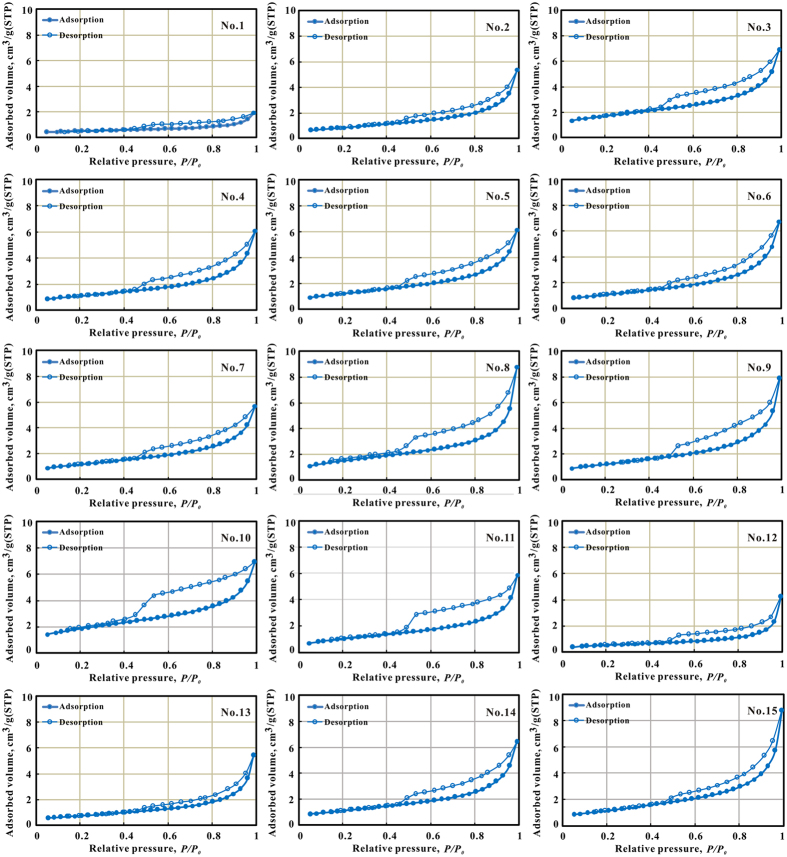
Adsorption-desorption isotherms for the 15 tight sandstones obtained at 77 K.

Nitrogen isotherms for selected tight sandstone samples were shown in Fig. 6. One point to note was that the samples used in N_2_GA analysis came from the same core as used for the grain size, mineral composition, porosity and MICP analysis. It can be noted that the adsorption-desorption process was not reversible, as observed by the presence of hysteresis loops. All the N_2_ isotherms obtained at 77 K were similar and did not strictly fall within the IUPAC classification group^21^. At the relative pressures (*P/P*_0_ greater than 0.45) the curve exhibited a hysteresis loop indicating the multilayer range associated with capillary condensation in mesopores (type IV). However, for these rocks, the horizontal plateau at relative pressures close to 1 did not occur. It is known that the isothermal shape still being hyperbolic at relative pressures close to 1 indicated that the material investigated also contained a range of macropores which cannot be analyzed by N_2_GA^20^,^38^.

Considering the shape of the hysteresis loops, we suggested that the tight sandstone rocks analyzed were mainly characterized by transitional form of type H2, type H3 and type H4. H2 shape hysteresis loop was often associated with pores that were characterized by narrow necks and wide bodies (ink-bottle pores), while H3 loop and H4 loop were observed with aggregates of plate-like particles giving rise to slit-shaped pores and narrow slit-like pore, respectively^21^,^39^. Some previous studies indicated that, for natural materials, such as seal or shale rocks, this interpretation had to be considered with caution since it was prone to error^38^. Such a caution was also echoed by Clarkson *et al*. (2012a) who studied tight gas sandstones using USANS/SANS and gas adsorption analysis^7^, and found that the assumption of slit-shape pores inferred from hysteresis loop shape was not consistent with the SANS scattering results. Nevertheless, pore shapes consistent with hysteresis loop included ink-bottle pores and slit-like pore associated with plate-like particles were indeed identified based SEM (Fig. 2I–L). It was worth noting that these pores were mainly intercrystalline pores developed in the clay minerals.

The nitrogen sorption measurements were used to evaluate the structures of pores in the range of 1.7–300 nm, such as their specific surface area, BJH volume and average pore size. Chang**-**7 tight sandstone had specific surface area values ranging from 1.73 to 6.56 m^2^/g, with an average of 3.99 m^2^/g (Supplementary Table S4). Their BJH pore volumes were in the range of 2.58–13.04 ml per 100 g, with an average of 9.13 ml per 100 g. The average pore size of the tight sandstone was 8.15–17.6 nm.

**Figure 7:**
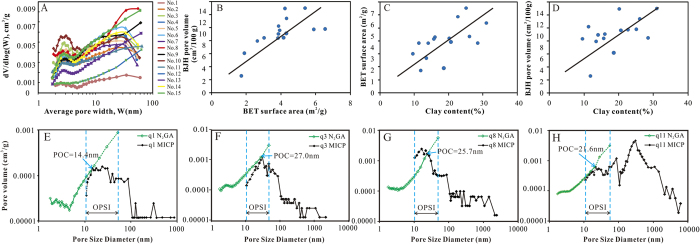
(**A**) Pore volume distribution with pore size derived from N_2_ adsorption branch for the isotherms of 15 samples using BJH model. (**B**) Positive correlation between BET surface area and BJH pore volume. (**C**) Positive correlation between clay content and BET surface area. (**D**) Positive correlation between clay content and BJH pore volume. Pore size distribution for (**E**) sample No. 1, (**F**) sample No. 3, (**G**) sample No. 8, and (**H**) sample No. 11 by MICP combined with N_2_GA are shown.

According to Groen *et al*.^23^, PSD derived from the desorption branch was often closely affected by pore network and always showed the artificial pores at approximately 4 nm. Thus, the adsorption branch was highly preferred for pore size calculations. The plot of d*V*/d*log(W*) (*V* = pore volume, *W* = pore width) versus *W* was frequently used to display the pore size distribution and was more conveniently used to compare the relative pore volumes among any pore size ranges^24^,^26^. These samples can be divided into two groups according to differences presented on pore size distribution determined by N_2_GA (Fig. 7A). One is characterized as unimodal (No.1 and No.12) with low BET surface area (1.73–1.9, with an average of 1.82 m^2^/g) and low BJH pore volume (2.58–6.14 with an average of 4.36 cm^3^/100 g). The other group was characterized as bimodal with sub peak around 2–4 nm and main peak around 35–60 nm, with high BET surface area (2.66–6.56 with an average of 4.32 m^2^/g) and BJH pore volume (7.99–13.04 with an average of 9.87 cm^3^/100 g). A positive correlation relationship existed between BJH pore volume and BET surface area (Fig. 7B), which was consistent with previous study in shale^40^,^41^. It was worth noting that BJH pore volume increased with the clay content (Fig. 7C) due to the fact that most of pores with sizes less than 300 nm were produced in clay mineral, as observed under SEM (Fig. 2I–L). As clay minerals were capable of adsorbing gas to their internal structure^30^, samples with more clay content had larger BET specific surface area (Fig. 7D).

### Pore size distribution determination using MICP combined with N_2_GA

As was mentioned above, both MICP and N_2_GA had measurement limitations in characterizing pore structure of tight oil reservoirs. To characterize the overall PSD of Chang-7 tight oil reservoirs, a combination of these two invasion methods was applied. Evidently there was an overlapping pore size interval (OPSI) with both techniques (Fig. 7E–H, interval between blue dashed lines), making it necessary to calculate the pore size at which the measurements of N_2_GA and MICP should be connected (point of connection-POC). The two techniques could be connected when filling pores had the same diameter and incremental volume, as described by Schmitt *et al*.^38^. The POC was at the first junction of the two curves, mainly located under 30 nm. Overall PSD of Chang-7 tight sandstones were broad and continuous from 2 nm to 10,000 nm (Fig. 7E–H).

## Discussion

**Figure 8:**
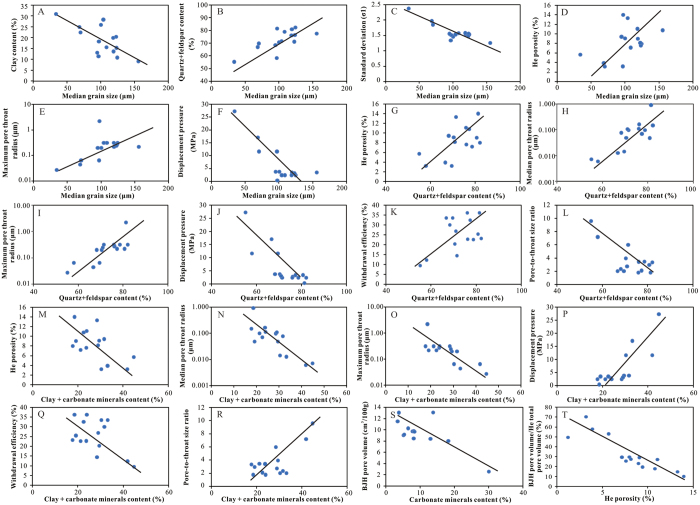
(**A**) Negative correlation between median grain size and clay content. (**B**) Positive correlation between median grain size and detrital mineral. (**C**) Negative correlation between median grain size and standard deviation (σ1). (**D**) Positive correlation betweenmedian grain size and He porosity. (**E**) Positive correlation between median grain size and maximum pore throat radius. (**F**) Negative correlation between median grain size and displacement pressure. Relationships between (**G**) detrital mineral content and porosity, (**H–L**) detrital mineral content and pore structure parameters, (**M**) interstitial material content and porosity, (**N–R**) interstitial material content and pore structure parameters, (**S**) carbonate mineral content and BJH pore volumes, (**T**) ratio of pore volume determined by N_2_GA (less than 300 nm) in total pore volume and porosity.

There was little doubt that grain size and mineral composition had effects on pore structure of Chang-7 tight sandstone as discussed in previous sections. Grain size of sediment was a significant symbol of depositional hydrodynamic conditions^36^. Sediments with coarser grain size were frequently formed in high energy depositional environments. These sediments typically possessed low clay content, high debris content and well sorted particles (Fig.8A–C). Sandy sediments with low matrix (Matrix is a sedimentology term referring to finer grained sedimentary materials such as clay or silt-scale minerals, in which larger grains or fragments are embedded) content tended to have more intergranular pores and relatively high original porosity^42^,^43^. Meanwhile, clay mineral had weaker compaction resistance than detrital minerals such as quartz and feldspar^42^. Well sorted sandstone also had stronger compaction resistance than poor sorted group. Thus, coarse grain size was one favorable factor for porosity and pore throat radius in the study area (Fig. 8D–F).

As for the mineral composition, samples with high detrital mineral content (quartz and feldspar) were often associated with high porosity and high quality pore structure. It was due to the fact that quartz and feldspar were framework mineral of sandstone and had strong compaction resistance. Furthermore, most of micron-sized pores in Chang-7 sandstones were relevant to feldspar dissolution in the study area. Detrital mineral content (DMC) was positively associated with porosity and pore throat radius while negatively related to displacement pressure (Fig. 8G–J). It was worth noting that DMC was positively associated with mercury withdrawal efficiency and negatively related to pore-to-throat size ratio (Fig. 8K–L). This finding suggested that high DMC content was in favor of throat development. This was because samples with high DMC content had less interstitial material, which may damage pores and shift pores to narrow throats.

Correlations between interstitial material content (clay and carbonate minerals) and pore structure parameters further supported this judgment. Interstitial material content (IMC) was negatively associated with porosity, pore throat radius and withdrawal efficiency while positively related to displacement pressure and pore-to-throat size ratio (Fig. 8M–R).

Clays and carbonate minerals played different roles in influencing porosity and pore structure. As mentioned in previous sections, clay content was positively correlated with BJH pore volume determined by N_2_GA (less than 300 nm), due to the amount of intercrystal pores produced in clay minerals. Carbonate minerals produced no pores, and presented even significantly negative correlation with BJH pore volume (less than 300 nm) (Fig. 8S). Actually, the ratio of pore volume determined by N_2_GA (less than 300 nm) in total pore volume of Chang**-**7 tight sandstone (ranging in 10.14–70.24% with an average of 32.32%) presented a downward trend with porosity rise (Fig. 8T). It meant that these nano-sized clay intercrystal pores did not play an important role in total porosity of Chang**-**7 tight sandstone, although some previous studies showed that clay pores held a dominant position in shale porosity^20^.

This study indicated that Chang-7 tight sandstone was characterized by fine grain, poorly sorted and mainly composed of detrital particles (quartz, feldspar and lithic fragment), carbonate cement (calcite and dolomite) and clay mineral. Chang-7 pore types mainly included micron-sized residue intergranular pores, dissolved pores, micro-fractures and nano scale clay inter-crystalline pores. Residue intergranular pores were produced in interval of framework grains (quartz, feldspar and lithic fragment) while dissolved pores were mainly related with dissolution of feldspar. Nano inter-crystalline pores existed widely in clay minerals. Coarse granularity was a favorable factor for both porosity and pore structure (e.g. pore throat radius, pore-to-throat size ratio and withdraw efficiency) in the study area. Coarser grain size meant higher detrital minerals content and better sorted, which both were beneficial to strong compaction resistance. As to mineral composition, quartz and feldspar was advantageous to porosity and pore structure while clay and carbonate cement were disadvantageous. It was due to the fact that quartz and feldspar constituted framework of Chang-7 sandstone producing intergranular pores while uppermost dissolved pores were mainly associated with feldspar. Beside this, quartz and feldspar had stronger compaction resistance. Although many intercrystalline pores developed in clay minerals, these nano-scale pores did not occupy leading position relative to other micro-sized pores (mainly feldspar dissolved pores). N_2_GA had its own limitation on pore structure study of tight sandstone limited by its measurement range (1.7–300 nm). MICP was more suitable for tight sandstone study, benefitted from its wider measurement ranges and obtained pore structure parameters. In spite of this, N_2_GA has its value to evaluate clay inter-crystalline pore in tight sandstone, including not only the pore volume, but also the pore shapes.

## Experimental Methods

### Microscopic observation, mineral composition, grain size and porosity analysis

Thin sections were made from more than 200 representative fresh and unweathered samples, and petrographic data were collected using a Nikon polarizing light microscope. Following information was provided: (1) framework grains, matrix and cement; (2) pore types. Quanta 200 F SEM (FEI, USA) was also used to examine pore morphology of freshly broken fragment samples. All samples were gold coated and placed for secondary electron imaging, backscatter electron imaging and energy dispersive spectroscopy mineral identification. The accelerating voltage and resolution of SEM were 30 kV and 1.2 nm respectively.

X-ray diffraction was performed to measure the relative content of each mineral composition by using X’Pert PRO MPD diffraction instrument (Panalytical, Netherlands): copper butt, pipe pressure 30 kV, conduit flow 40 mA, scanning speed 2°(2θ)/min. The sample was grounded to grains of 320-mesh, and the natural thin section was made tomeasure and analyze the relative content of each mineral.

As to grain sizes, samples were successively pre-treated with 10 ml 30% H_2_0_2_ to remove organic matter, with 5 ml 10% HCl to remove calcium carbonates, and with 300 mg of Na_4_P_2_O_7_·10H_2_O to further disperse grains^44^. The grain size distribution was measured by a Mastersizer 2000 laser diffraction instrument (Malvern Instruments Ltd, UK) with 100 bins ranging from 0.02 to 2000 μm. Core samples porosities were measured using an Ultrapore-200A helium core porosimeter (Core Lab, USA).

### Mercury injection capillary pressure (MICP)

The MICP method was based on the fact that mercury behaved as a non-wetting liquid when in contact with most solids. Consequently, it did not penetrate into the openings and cracks of these substances without the application of pressure. The pressure (*P*_*w*_) required for mercury to penetrate pores was a function of the contact angle (*θ*_*Hg*_) of mercury with the porous material to be intruded, its gas/liquid surface tension (*γ*_*Hg*_) and pore radius (*r*_*p*_). This relationship was given by the Young-Laplace law for the particular case of cylindrical pores as the Washburn equation^45^:


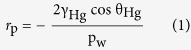


Eq. (1) indicated that with increasing pressure, the mercury will intrude into progressively narrower pores for constant values of *γ*_*Hg*_ and *θ*_*Hg*_. The volume of mercury (*V*) penetrating the pores was measured directly as a function of applied pressure. This *P*-*V* data provided a unique characterization of the pore structure. The pore wall surface obtained from MICP data was computed on the basis of the reversible work (d*W* = *P*d*V*) required to immerse an area (*dS*) of a non-wetting surface in mercury as^46^,


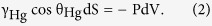


Assuming that *γ*_*Hg*_ and *θ*_*Hg*_ did not vary with pressure, for the evaluation based on the *P*-*V* mercury penetration data Eq. (2) became,


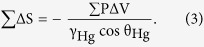


The mercury porosimetry experiments were carried out with a PORE SIZER 9320 (Micromeritics Instrument Corp., USA). The equivalent pore radius was computed according to the capillary pressure using the Washburn equation (1) with *P*_*w*_ ranging from 0.003 to 136 MPa, using a contact angle of 130°^27^ and surface tension of 485 dyne/cm^47^.

### Low-pressure nitrogen (N_2_) gas adsorption (N_2_GA)

Low pressure (less than 0.127 MPa, 77.35 K) N_2_ sorption analyses were performed using a Tristar II 3020 surface area analyzer (Micromeritics Instrument Corp., USA) at State Key Laboratory of Heavy Oil Processing in China University of Petroleum, Beijing.

N_2_ adsorption analysis was measured at 77.35 K which was lower than the N_2_ critical temperature (190.6 K). Therefore, pores would undergo two stages of adsorption, firstly with single and multi-layer adsorption, and secondly with capillary condensation^48^. Traces of gas and water molecules available in the sample competed with the nitrogen molecules for adsorption sites, therefore, it was required to remove moisture content and to degas the samples prior to pore structure analysis^49^. For drying the shale samples, the samples were oven dried for 8 hours at 110 °C similar to the preparation procedure for gas expansion method. It can be used to obtain the following information in microporous materials^27^: (1) specific pore volume: pore volume (1.7–300 nm) per mass of the sample expressed as cm^3^/g, (2) shape of the pores, (3) specific surface area: total surface area per mass of the sample expressed as m^2^/g, and (4) pore sizes and their distribution.

In the following section, we provided a brief explanation on the theory behind the extraction of pore volume and pore size distribution based on the results of low pressure adsorption measurement.

The adsorption measurement was used to quantify the amount of gas adsorbed at different relative pressures (*P/P*_0_) where *P* was the gas vapor pressure in the system and *P*_0_ was the saturation pressure of adsorbent. Micromeritics instrument gave the adsorption isotherm point by point by measuring quantity of nitrogen adsorbed and the equilibrium pressure. Desorption isotherm can be obtained by measuring the quantities of gas removed from the sample as the relative pressure was lowered. The total pore volume was derived from the amount of vapor adsorbed at relative pressure close to unity, by assuming that the pores werethen filled with liquid adsorbate. The classical pore size model developed by Barret *et al*.^22^, which was based on the Kelvin equation and corrected for multilayer adsorption, was most widely used for calculation of PSD with an upper pore diameter limit of ~300 nm^18^,^25^.

The BJH analysis described the capillary condensation phenomenon in a cylindrical pore. It was assumed that the condensation of a fluid in a pore of radius *r*_0_ took place in the ‘core’ region, i.e., the inner part of the pore that had a radius *r*_0_-*t*(*P/P*_0_), where *t*(*P/P*_0_) was the thickness of the film adsorbed on the pore wall as a function of the relative pressure (*P/P*_0_) of the gas. Using this model, it was predicted that the condensation of nitrogen in a pore of radius *r*_0_ occurred at a pressure given by the following modified Kelvin equation:


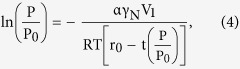


where 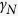 and *V*_1_ were the surface tension and molar volume of liquid nitrogen, respectively, *R* was the gas constant, *T* was the absolute temperature at which the isotherm was measured (77 K) and 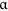 was a factor that accounted for the shape of the gas/liquid interface. The latter was assumed to be cylindrical during the adsorption process (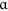 = 1) and hemispherical during the desorption process (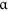 = 2)^50^. In this study, *t* was determined using the empirical Halsey equation for nitrogen^51^.


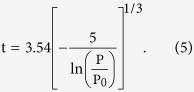


All the measurements were done at the State Key Laboratory of Petroleum Resources and Prospecting, associated with the China University of Petroleum, Beijing.

## Additional Information

**How to cite this article**: Cao, Z. *et al*. Pore structure characterization of Chang-7 tight sandstone using MICP combined with N_2_GA techniques and its geological control factors. *Sci. Rep.* **6**, 36919; doi: 10.1038/srep36919 (2016).

**Publisher’s note:** Springer Nature remains neutral with regard to jurisdictional claims in published maps and institutional affiliations.

## Supplementary Material

Supplementary Information
