# Supplementary material for: Pore structure characterization of Chang-7 tight sandstone using MICP combined with N2GA techniques and its geological control factors
Source: Sci Rep. 2016 Nov 10;6:36919. doi: 10.1038/srep36919 (PMC5103269; doi:10.1038/srep36919)
Supplement: Supplementary Information [file srep36919-s1.doc]

# Pore structure characterization of Chang-7 tight sandstone using MICP combined with N_2_GA techniques and its geological control factors

Zhe Cao^1, 2^, Guangdi Liu^1, 2^,*, Hongbin Zhan^3^, **, Chaozheng Li^1, 2^, Yuan You^4^, Chengyu Yang^1, 2^, Hang Jiang^5^

^1^State Key Laboratory of Petroleum Resources and Prospecting, China University of Petroleum, Beijing 102249, PR China

^2^ College of Geosciences, China University of Petroleum, Beijing 102249, PR China

^3^Department of Geology and Geophysics, Texas A&M University, College Station, TX 77843-3115

^4^Research Institute of Petroleum Exploration and Development of Changqing Oilfield Company, PetroChina, Xi’an 710018, PR China

^5^Strategic Research Center of Oil and Gas Resource, Ministry of Land and Resources, Beijing 100034, PR China

* Corresponding author.

E-mail address: [guangdiliucupb@gmail.com](mailto:guangdiliucupb@gmail.com) (G. D. Liu)

** Corresponding author.

E-mail address: [zhan@geos.tamu.edu](mailto:zhan@geos.tamu.edu) (H. B. Zhan)

## Supplementary Information

**Supplementary Table S1**. Mineral composition identified based on XRD analysis and porosity of Chang-7 sandstone in study area.

**Supplementary Table S2**. Summary of grain size parameters: D(0.05), D(0.16), D(0.25), D(0.50), D(0.75), D(0.84), D(0.95), median, ,mean and standard deviation index for Chang-7 sandstones in study area.

**Supplementary Table S3**. Summary of the pore structure data and parameters derived from MICP analysis for the Chang-7 tight sandstone samples. *So*: sorting coefficient; *P_c_*_50_: median capillary pressure; *r*_50_: median pore throat radius; *P_d_*: displacement pressure; *r_max_*: maximum pore throat radius; *S_max_*: maximum mercury saturation; *We*: mercury withdrawal efficiency.

**Supplementary Table S4**. BET surface area, total pore volume (1.7-300nm) and average pore diameter of Chang 7 Tight sandstone from N_2_GA.
